# Precocious infant fecal microbiome promotes enterocyte barrier dysfuction, altered neuroendocrine signaling and associates with increased childhood obesity risk

**DOI:** 10.1080/19490976.2023.2290661

**Published:** 2023-12-20

**Authors:** Germaine J. M. Yong, Cara E. Porsche, Alexandra R. Sitarik, Kei E. Fujimura, Kathryn McCauley, Dat T. Nguyen, Albert M. Levin, Kimberley J. Woodcroft, Dennis R. Ownby, Andrew G. Rundle, Christine C. Johnson, Andrea Cassidy-Bushrow, Susan V. Lynch

**Affiliations:** a Division of Gastroenterology, Department of Medicine, University of California, San Francisco, CA, USA; b Asian Microbiome Library Pte Ltd, Singapore and Singapore Institute of Food and Biotechnology Innovation, Singapore, Singapore; c Department of Public Health Sciences, Henry Ford Health System, Detroit, MI, USA; d Genetic Disease Laboratory, California Department of Public Health, San Francisco, CA, USA; e Department of Molecular Microbiology and Immunology, Johns Hopkins Bloomberg School of Public Health, Baltimore, MD, USA; f Division of Allergy and Clinical Immunology, Department of Pediatrics, Augusta University, Augusta, GA, USA; g Department of Epidemiology, Mailman School of Public Health, Columbia University, New York, NY, USA

**Keywords:** Early life, gut microbiome, nutrition, childhood obesity, gut barrier dysfunction

## Abstract

Early life gut microbiome composition has been correlated with childhood obesity, though microbial functional contributions to disease origins remain unclear. Here, using an infant birth cohort (*n* = 349) we identify a distinct fecal microbiota composition in 1-month-old infants with the lowest rate of exclusive breastfeeding, that relates with higher relative risk for obesity and overweight phenotypes at two years. Higher-risk infant fecal microbiomes exhibited accelerated taxonomic and functional maturation and broad-ranging metabolic reprogramming, including reduced concentrations of neuro-endocrine signals. *In vitro*, exposure of enterocytes to fecal extracts from higher-risk infants led to upregulation of genes associated with obesity and with expansion of nutrient sensing enteroendocrine progenitor cells. Fecal extracts from higher-risk infants also promoted enterocyte barrier dysfunction. These data implicate dysregulation of infant microbiome functional development, and more specifically promotion of enteroendocrine signaling and epithelial barrier impairment in the early-life developmental origins of childhood obesity.

## Introduction

One in three children in the United States experience overweight or obesity [defined as body mass index (BMI) ≥85th percentile for age and sex] and, as a result, are at increased risk of co-morbidities including heart and fatty liver disease, diabetes, and asthma.^1^ In adults, gut microbiome perturbation is characteristic of patients with obesity^2–5^ and its functional contribution to disease pathogenesis has been confirmed in humanized gnotobiotic mouse models.^6,7^ Several murine and human studies have correlated early-life gut microbiota perturbation with subsequent adiposity during adolescence and adulthood.^8–13^ However, none have provided data for a functional role of the human infant gut microbiome in the subsequent development of childhood obesity or identified infant microbiomes features that promote obesogenic responses.

Early-life gut microbiome development progresses along a temporal gradient.^14–16^ Alterations to the composition and rate of microbial accumulation during this critical developmental window are related to risk of childhood conditions that impact body size, including impaired growth^13,17^ and Kwashiorkor (protein malnutrition).^18–20^ Diet plays a significant role in shaping the gut microbiome,^19^ which in turn influences the metabolic fate of ingested nutritional substrates and the production of bioactive molecules that influence host cell physiology.^20–23^ Mode of delivery and nutrition in infancy have been associated with alterations to the infant gut microbiome and with obesity in childhood,^24,25^ though the mechanisms by which they contribute to disease remain unclear. Here, to better understand the origins of childhood obesity, we focus on very-early infancy and identify a compositionally and functionally precocious fecal microbiome at one month of age associated with correlates of lower socioeconomic status, decreased rates of exclusive breastfeeding and with heightened risk for overweight or obese phenotypes (OW/OB) in early childhood. We provide evidence for how the infant fecal microbiome may functionally contribute to childhood obesity, including promoting expansion of nutrient sensing enteroendocrine progenitor cells, induction of enterocyte transcriptional programs characteristic of obesity and increasing epithelial barrier dysfunction. Thus, our data suggest that early-life nutrition and appropriately paced microbiota functional and metabolic development appear crucial determinants of childhood obesity development.

## Results

### Distinct infant fecal microbiota associates with OW/OB at age two years

**Figure 1.**
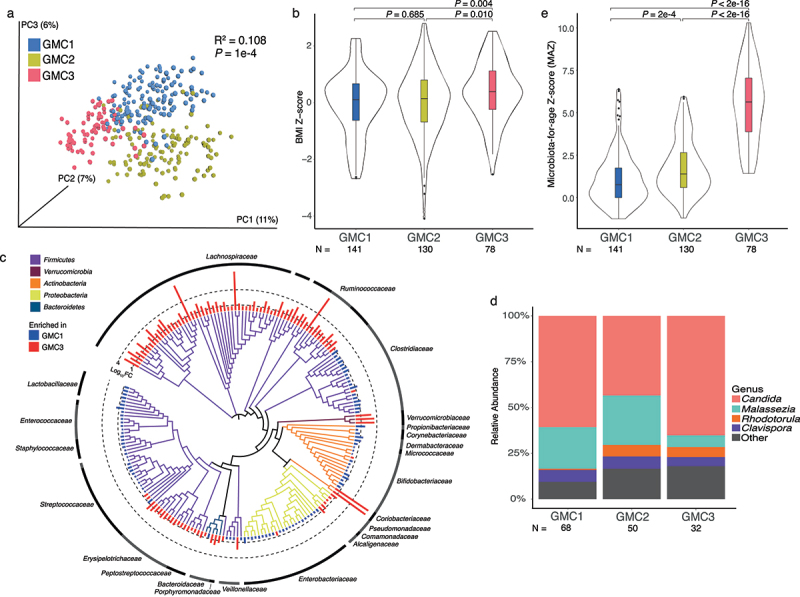
Compositionally distinct gut microbiota classes (GMCs) in feces of 1-month-old infants exhibit differential microbiota maturity and relate to the relative risk (RR) of overweight/obesity (OW/OB) at age 2 years.

Fecal samples collected in early postnatal life (*n* = 349; median age 35 days; range 21–58 days) were subjected to parallel 16S rRNA and ITS2 sequencing. Breastfeeding, delivery mode, parity, maternal BMI and smoke exposure during pregnancy and various factors reflective of parental socioeconomic status explained a significant proportion of the variance observed in the fecal microbiota profiles across infants (PERMANOVA, p_all_ <0.05; Supplementary Table S1). Dirichlet Multinomial Mixture (DMM) modeling, which implements an unsupervised Bayesian approach to class discovery,^26^ was applied to the 16S rRNA dataset to classify participants based on fecal bacterial community composition. Three distinct gut microbiota classes [GMC1 (*n* = 141/349), GMC2 (*n* = 130/349), and GMC3 (*n* = 78/349)] represented the best model fit (Unweighted UniFrac; PERMANOVA; R^2^ = 0.11; *p* = 1e^−4^; Figure 1a and Supplemental Figure S1a). Notably, GMC3 infants were more likely to have unmarried mothers with a shorter duration of formal education and to have been exclusively formula fed, while GMC1 infants had the greatest proportion of exclusively breastfed infants (Supplementary Table S2). BMI Z-scores of GMC3 infants were greater than either GMC1 (β = 0.51; 95% CI 0.17–0.85; *p* = 0.004; Figure 1b and Supplementary Table S3a) or GMC2 groups (β = 0.45; 95% CI 0.11–0.79; *p* = 0.010; Figure 1b and Supplementary Table S3a) after adjusting for variables known to be related to both microbiome composition and obesity (i.e. age at stool sample collection, maternal BMI at first measure during pregnancy, prenatal antibiotic use, prenatal antifungal use, mode of delivery, and breastfeeding status at 1-month) and which resulted in a change-in-effect estimate of the GMC and body size association (Supplementary Table S3a and Supplemental Figure S1b-c). Relative risk (RR) for OW/OB at age two was also greater for GMC3 compared with GMC1 infants (Adjusted Multivariable Model; RR = 2.52; 95% CI 1.33–4.79; *p* = 0.005; Supplementary Table S3b and Table 1). This represented an 8% increase in risk over the unadjusted RR (Supplementary Table S3b), indicating that the observed relationship between the infant fecal microbiota and childhood OW/OB persists and is in fact strengthened when factors known to be related to these outcomes are adjusted for in statistical models.

(a) Distinct GMCs identified by Dirichlet Multinomial Mixture modeling explain more than 10% of observed variation in infant fecal bacterial β-diversity (*n* = 349; PERMANOVA of Unweighted UniFrac distances). (b) BMI Z-score at age 2 years significantly differs between infants with distinct GMCs; In multivariate models adjusted for several confounding factors, GMC3 infants exhibit significantly higher BMI Z-scores in childhood. (c) Bacterial taxonomic comparison of GMC3 and GMC1 subjects; taxa exhibiting significant differences (zero-inflated negative binomial regression (ZINB); P_FDR_ < 0.05) in mean relative abundance are shown. Values are log_10_-transformed for purposes of illustration. Bar height indicates the magnitude of between-group difference in relative abundance. Bacterial phyla are color coded as indicated. (d) Relative abundance of fungal genera differs across GMCs. (e) Microbiota-for-age Z-scores (MAZ) of higher-risk GMC3 infants are significantly greater than that of lower-risk infants (*n* = 349; Kruskal-Wallis; *P* < 2e-16), indicating accelerated microbiota maturation in high-risk infants. MAZ scores were calculated from a random forest model trained on the 50 most age-discriminatory bacterial OTUs of normal BMI infants. Pairwise comparisons were calculated using two-sided Wilcoxon rank sum tests in c. Boxplots indicated within violin plots represent the median (center), the 25^th^ and 75^th^ percentiles, and the smallest and largest values within 1.5 × the interquartile range (whiskers).

**Table 1.** Infant gut microbiota classes (GMCs) exhibit significantly different relative risk ratios (RRs) of developing OW/OB phenotypes at age 2 years.

	Microbial Community Types			RR^1^ (95% Cl)		
	GMC1(*N* = 141)	GMC2 (*N* = 130)	GMC3 (*N* = 78)	GMC3 vs. GMC1	GMC3 vs. GMC1	GMC3 vs. GMC2
Overweight or Obese at Age 2	16 (10%)	25 (19%)	21 (27%)	2.52 (1.33–4.79) *P* = *0*.*005*	1.60 (0.85–3.03) *P* = *0*.*146*	1.57 (0.91–2.73) *P* = *0.105*

^1^Adjusted for age (in days) at stool sample collection, maternal BMI at first measure during pregnancy, prenatal antibiotic and antifungal use, mode of delivery and breastfeeding status at 1-month. Between-group risk ratio significance values were calculated using log-binomial regression.

DMM modeling applied to older infant samples (median age 206 days; range 174–238 days; *n* = 287) identified four distinct fecal microbiota structures (GMC4–7; Unweighted UniFrac; PERMANOVA, R^2^ = 0.15, *p* = 0.001; Supplemental Figure S1d-e), however these were not significantly related to BMI Z-score or OW/OB phenotypes at age two years (Supplementary Table S4a-b). This may be due to reduced power to detect such associations attributable to increased microbiota compositional variation associated with environmental exposures, breastfeeding cessation and dietary diversification at this later life stage.^15,27^

### Infants at higher risk for OW/OB exhibit precocious fecal microbial development

Despite exhibiting increased bacterial species richness (*p* < 2e-16, Supplemental Figure S1f) and phylogenetic diversity (*p* < 2e-16, Supplemental Figure S1g) compared with GMC1 or GMC2 microbiota, GMC3 communities were depleted of key genera (e.g. *Bifidobacterium*, *Clostridium* and *Malassezia*) typical of this stage of postnatal development^14,28^ (Figure 1c–d and Supplemental Figure S2a-b and Supplementary Tables S5 and S6). Instead, they exhibited relative enrichment of *Lachnospiraceae* (including *Blautia, Ruminococcus* and *Dorea*), *Ruminococcaceae* (including *Oscillospira* and *Faecalibacterium*) and *Saccharomyces* and *Candida* (Figure 1c–d and Supplementary Tables S5 and S6), genera typically associated with later stages of infant gut microbiome development^14,28^ (Supplemental Figure S2a-b) and, in adult populations, with greater visceral fat mass^4,29,30^ and insulin resistance.^4^ These microbial enrichments were consistent irrespective of the lower-risk comparison group used (Supplementary Tables S7 and S8) and included several age-discriminatory bacterial taxa identified through random forest predictive modeling of healthy infants (Supplemental Figure S2c-e) that are associated with more mature infant gut microbiomes.^14,31^ Consistent with this observation, GMC3 infants exhibited older microbiota-for-age z-scores [Microbiota-for-age Z-score (MAZ); *p* < 2e-16; Figure 1e], indicating precocious early life intestinal microbiota development in infants at higher-risk for OW/OB in early childhood.

### Higher-risk GMC3 infant microbiomes exhibit broad metabolic and functional reprogramming

**Figure 2.**
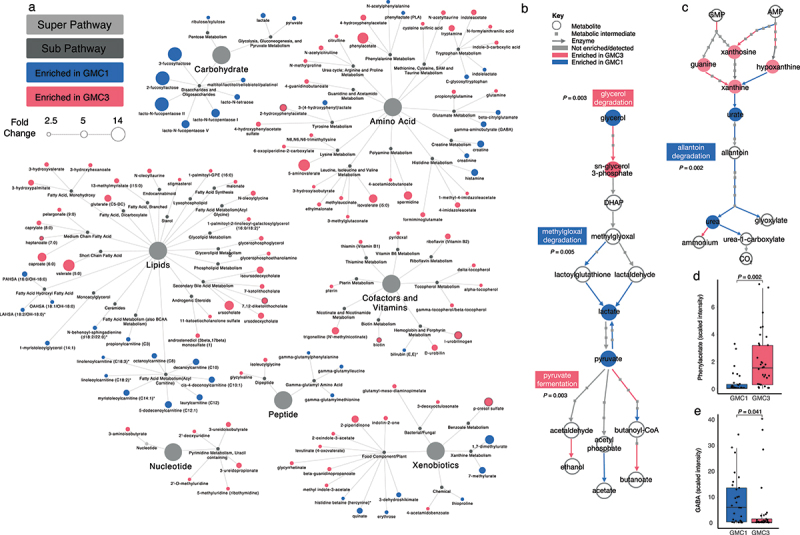
High-risk GMC3 and lower-risk GMC1 exhibit distinct metabolic productivity and functional capacities.

To determine whether the compositionally distinct GMC3 fecal microbiota associated with increased OB/OW risk produced a distinct suite of metabolites from that of their lower risk counterparts, untargeted mass spectrometry was performed on a subset of infant feces (*n* = 60) chosen based on highest posterior probability of GMC1 or 3 membership (*P* > 0.90) and a balanced distribution of BMI classifications (i.e. per GMC, approximately equal numbers of infants who developed normal BMI and OW/OB phenotypes at age two years; Supplementary Table S9). Comparative analyses evidenced distinct metabolic productivity between lower- and higher-risk GMCs (Figure 2a and Supplementary Table S10), particularly within the lipid (short and medium chain fatty acid, secondary bile acid and glycerophospholipid classes), amino acid (aromatic and branched chain) and vitamin and co-factor (B and E vitamins and biotin) classes of metabolites. In several cases, these metabolic differentials were corroborated by analysis of targeted pathways in paired shotgun metagenomic data derived from the same fecal samples, indicating that several of the metabolites associated with childhood obesity risk are most likely microbially derived products. GMC3 feces were enriched both for glycerol 3-phosphate (*P*_*FDR*_ = 0.05; Figure 2b and Supplemental Figure S3a), a metabolite involved in glycolysis and mitochondrial oxidative phosphorylation, and for the microbial glycerol degradation pathway responsible for its production (*P* = 0.003; Figure 2b, Supplemental Figure S4a). GMC1 feces were enriched for lactate and pyruvate (*P*_*FDR*_ = 0.047 and *P*_*FDR*_ = 0.029 respectively; Figure 2b and Supplemental Figure S3b-c), products of the methylglyoxal detoxification pathway which was also found to be significantly enriched in paired fecal metagenomes of GMC1 infants (*P* = 0.005; Figure 2b and Supplemental Figure S4b). In addition, the significant depletion of pyruvate observed in GMC3 feces was paralleled by an increase in abundance of microbial encoded pathways for pyruvate fermentation (*P* = 0.003 for both; Supplemental Figure S4c-d), though products of these pathways were not observed in our liquid chromatography mass spectrometry data, plausibly due to their volatility.

(a) Comparison of fecal samples from GMC3 (*n* = 32) and GMC1 (*n* = 28) by untargeted mass spectrometry identifies significant (*P*_*FDR*_ < 0.05) widespread metabolic differences. Many metabolic differences between GMCs corroborate with differential expression of targeted pathways identified in paired shotgun metagenomic data, including (b) glycerol and methyglyoxal degradation, pyruvate fermentation and (c) allantoin degradation. In b and c, enzymes (arrows) and metabolites (open circles and closed squares) with log_2_ FC > |0.25| are color coded by GMC enrichments (red enriched in GMC3; blue enriched in GMC1). (d) Phenylacetate and (e) GABA levels differ between GMC3 and GMC1. Differences in normalized abundance between groups determined by zero-inflated compound Poisson (ZICP) regression (Supplementary Table S10a). In d and e, significance was calculated using Welch’s two-sided t-test, with P_FDR_ < 0.05 considered significant, and each dot represents an independent infant stool sample. Boxplots represent the median (center), the 25th and 75th percentiles, and the smallest and largest values within 1.5 × the interquartile range (whiskers).

Lower-risk GMC1 fecal metabolomes exhibited significantly elevated concentrations of human milk oligosaccharides (2-fucosyllactose, 3-fucosyllactose, lacto-N-fucopentaose and lacto-N-tetraose; Figure 2a and Supplementary Table S10). GMC1 was also enriched in a large range of distinct lipids, including fatty acid esters of hydroxy fatty acids (FAHFAs) and acylcarnitines (Figure 2a and Supplementary Table S10), the latter indicative of enhanced capacity for mitochondrial fatty acid β-oxidation. While GMC1 exhibited relatively higher concentrations of bilirubin (*P*_*FDR*_ = 0.021; Supplemental Figure S3d), GMC3 was enriched for the microbially reduced heme catabolic product, urobilinogen (*P*_*FDR*_ = 0.011; Supplemental Figure S3e), which is associated with obesity^32^ and older age.^33^

Higher-risk GMC3 feces exhibited significantly increased concentrations of several vitamin and amino acid derivatives (Figure 2a and Supplementary Table S10) despite being depleted of microbial pathways responsible for their production (≤*P* = 0.01 for all; Supplemental Figure S4e-h), and for the metabolism of allantoin (*P* = 0.002; Figure 2c and Supplemental Figure S4i) which recycles nitrogen necessary for their synthesis. Such dichotomy may reflect a dietary surplus of these vitamin and amino acids in higher-risk infants who were more likely to be formula fed. GMC3 feces were highly enriched for a range of catabolic amino acid by-products (Figure 2a), including phenylacetate (*P*_*FDR*_ = 0.002; Figure 2d) which is known to trigger hepatic steatosis,^34^ and modulators of GABAergic signaling, such as the gamma-aminobutyrate acid (GABA) precursor glutamine (*P*_*FDR*_ = 0.006; Supplemental Figure S3f) and weak GABA agonist 5-aminovalerate (*P*_*FDR*_ = 0.024; Supplemental Figure S3g). Notably, GABA, which regulates neuro-endocrine body weight control,^35,36^ obesity-induced inflammation,^37^ insulin sensitivity^37^ and intestinal epithelial barrier integrity,^38,39^ was depleted in the feces of higher-risk GMC3 infants (*P*_*FDR*_ = 0.041; Figure 2e). These latter observations implicate altered enteroendocrine signaling in higher-risk for obesity infants.

### Cell-free products of higher-risk infant fecal microbiomes promote transcriptional and functional reprogramming of enterocytes and reduce intestinal barrier integrity

**Figure 3.**
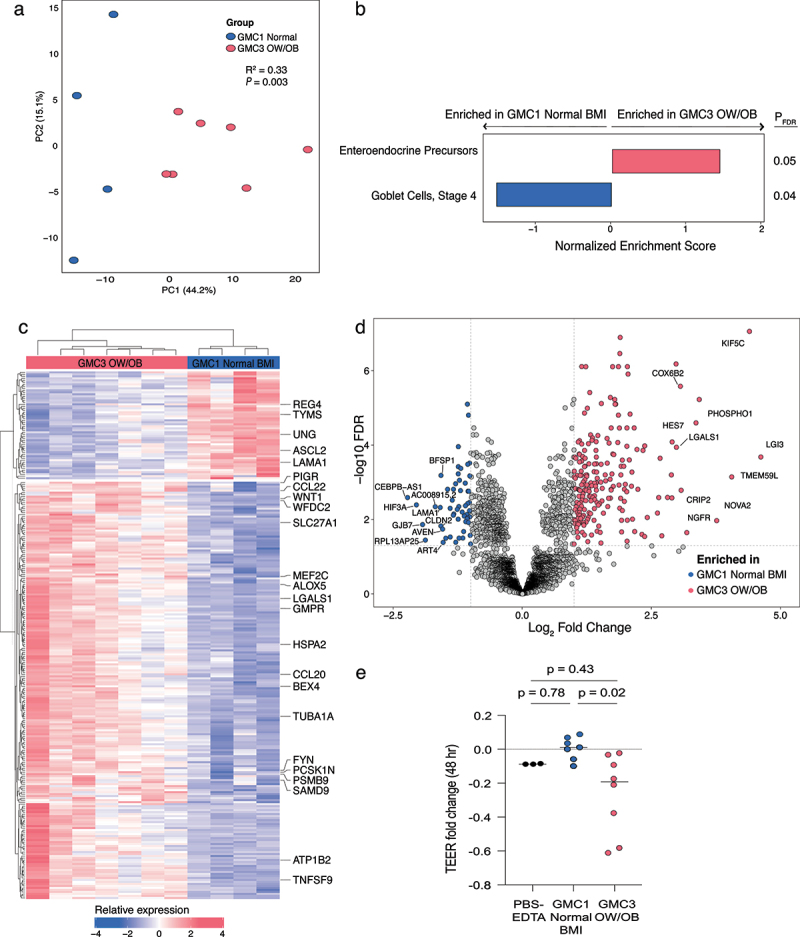
Cell-free fecal products from GMC3 infant microbiomes who developed OW/OB phenotypes in childhood reprogram Caco-2 enterocyte transcription.

The intestinal epithelium regulates nutrient uptake, inflammation, and intestinal barrier integrity and is constantly exposed to diffusible small molecules secreted by the fecal microbiome. Hence, we examined the effect of cell-free fecal microbiome products of metabolically profiled GMC3 and GMC1 infants (*n* = 17 samples with sufficient feces remaining for assay) on the transcriptional response and differentiation of Caco-2 enterocytes in the presence of oleic acid, the most abundant unsaturated fatty acid found in human and formula milk.^40^ Variance in enterocyte transcriptional response associated with GMC (Euclidean; PERMANOVA; R^2^ = 0.18; *p* = 0.005; Supplemental Figure S5a) and OW/OB status (Euclidean; PERMANOVA; R^2^ = 0.12, *p* = 0.046; Supplemental Figure S5b) and was greatest between the extremes of microbiome and obesity status [i.e. GMC3 OW/OB (*n* = 7) and GMC1 Normal BMI (*n* = 4); Supplemental Figure S5c]. This latter comparison produced starkly different transcriptional programs (Euclidean; PERMANOVA; R^2^ = 0.33, *p* = 0.003; Figure 3a and Supplementary Table S11). To assess potential cell types involved in these responses we applied gene set enrichment analysis, and found genes associated with enteroendocrine progenitors to be enriched in enterocytes treated with GMC3 OW/OB extracts (*P*_*FDR*_ = 0.05; Figure 3b). This is consistent with our observation that GMC3 microbiomes were enriched in metabolic products with the potential to alter enteroendocrine function and is further supported by the observation that *PCSK1N*, which controls neuroendocrine peptide precursor proteolysis,^41^ was also upregulated in this context (Figure 3c). Genes associated with mucin-producing goblet cells were enriched in enterocytes treated with fecal extracts from GMC1 infants who had normal BMIs at age two (*P*_*FDR*_ = 0.04, Figure 3b), indicating that microbiome-induced gastrointestinal mucus production, a critical physical and biochemical barrier, may play a protective role against childhood obesity development. GMC3 OW/OB fecal extracts also increased enterocyte expression of genes regulating inflammation (*ALOX5*, *TNFSF9*, *CCL20*, *CCL22*; Figure 3c), cell proliferation (*LGALS1*, *WNT1*; Figure 3c,d) and long-chain fatty acid transport (*SLC27A1*, *ACSL1*, *ACSL3*, *ACSL4*; Figure 3c and Supplemental Figure S5d), as well as those regulating vesicle maturation, transport and exocytosis (*KIF5C*, *LGI3*, *TMEM59L*; Figure 3d). In parallel, decreased expression of genes regulating barrier function (*GJB7*, *CLDN2*; Figure 3d), cellular organization (*BFSP1*, *LAMA1*; Figure 3d), fatty acid oxidation (Supplemental Figure S5d) and mitochondrial oxidative phosphorylation (Supplemental Figure S5e) were also observed. These data implicate the metabolic products of GMC3 infant fecal microbiomes in the induction of altered programs of enterocyte lipid trafficking and metabolism and in the promotion of diminished epithelial barrier integrity.

(a) GMC and OW/OB sample groupings explain a large proportion (33%) of observed variance in Caco-2 enterocyte response to cell-free fecal products of 1-month-old infants (Blue = GMC1 normal BMI, *n* = 4; Red = GMC3 OW/OB, *n* = 7; PERMANOVA of Euclidean distances). (b) Gene-set analysis of transcripts associated with intestinal epithelial cell states (Parikh *et al*. 2019) indicate enrichment of enteroendocrine precursors in response to cell-free fecal products of GMC3 OW/OB infants. (c) Heat map and (d) volcano plot of significantly (P_FDR_ < 0.05) differentially (log_2_ FC > |1|) expressed genes observed following exposure of enterocytes to cell-free fecal products of GMC3 OW/OB (*n* = 7) or GMC1 Normal BMI (*n* = 4) infants. Genes associated with obesity phenotypes and distinct intestinal epithelial cell types (Parikh *et al*. 2019) are labeled in c. (e) Cell-free fecal products of GMC3 OW/OB (*n* = 8) infants reduce transepithelial electrical resistance (TEER) of Caco-2 cells following 48 hours of treatment compared with cell-free fecal products from GMC1 normal BMI (*n* = 7) infants (One-way ANOVA; *P* = 0.02). PBS-EDTA represents control exposure. For a-e, n indicates biologically independent infant samples. Each dot represents one independent infant fecal sample in a and e and one transcript in d. DESeq2 was used to calculate significant genes using a two-sided FDR and log_2_ FC.

To validate these observations, we next tested the effect of GMC1 or GMC3 luminal products on epithelial barrier integrity. Polarized Caco-2 cell trans-well cultures were exposed to cell-free fecal extracts and transepithelial electrical resistance (TEER) was assessed pre- and post-exposure, using a subset of samples (*n* = 15) that had also undergone untargeted metabolomic analysis. Compared to cell-free products from GMC1 infants who developed normal BMIs, GMC3 OW/OB extracts were associated with reduced barrier integrity, a phenotype that was evident at 24 hours (*p* = 0.093; Supplemental Figure S6a) and significantly exacerbated by 48 hours post-exposure (*p* = 0.02; Figure 3e). Since increased intestinal permeability is often accompanied by inflammation in the context of obesity, we also assessed whether cell-free fecal products induced inflammatory cytokine secretion by Caco-2 cells. IL-1β, IL-8, and CCL20 are among the few cytokines produced by enterocytes in the absence of crosstalk from immune cells.^42^ ELISA-based quantification of these cytokines in basolateral supernatants resulted in minimal or no detection in any experimental group (Supplemental Figure S6b-d), suggesting that epithelial cross-talk with immune or microbial cells may be necessary to induce inflammatory responses associated with increased obesity risk.

To identify potential mediators of microbiome-derived epithelial permeability, we assessed associations between TEER values and microbiota composition and metabolic profiles. Fecal bacterial composition significantly related to TEER values (Bray-Curtis; PERMANOVA; R^2^ = 0.14, *p* = 0.02; Supplemental Figure S6e). To identify specific bacteria implicated in regulation of epithelial integrity, fixed effects models of taxon relative abundance were generated for samples used in the TEER assay. This analysis identified *Lactobacillaceae, Staphylococcaceae, Bacteroidaceae, Bifidobacteriaceae*, and *Clostridiaceae* to be associated with increased epithelial integrity while members of *Enterobacteriaceae* and *Streptococcaceae* related to increased barrier permeability (*P_FDR_* < 0.05 for all; Supplemental Figure S6f). Although variance in fecal metabolomes did not correlate with epithelial hyperpermeability (Euclidean; PERMANOVA; R^2^ = 0.84, *p* = 0.21), several individual metabolites were associated with TEER values (Spearman correlation; *P*_*FDR*_ < 0.06, Supplemental Figure S6g). Specifically, 2-piperidinone was associated with decreased epithelial integrity, while acyl carnitines, hydroxyhippurate, creatine and agmatine correlated with improved intestinal barrier integrity.

## Discussion

Early-life microbiome development is the product of evolutionary selection and dynamic functional synergy with the human host in the context of environmental exposures. Emerging evidence indicates that alterations to the rate of intestinal microbiome development disrupts the chronological synchrony of microbial-host interactions, impacting microbiome assembly, productivity and early-life developmental programming, resulting in childhood disease.^17,18,43,44^ Previously, a Canadian birth cohort uncovered an association between 3-month-old gut microbiota composition and risk of OW/OB at 12-months of age.^10^ Additional studies^13,45–51^ have similarly examined the relationship between the early life microbiota and future risk of obesity, and our findings largely agree with previous investigations. For example, *Collinsella*, *Parabacteroides* and members of the *Ruminococcaceae* family, which are enriched in infants at greater risk of childhood OW/OB in our study, have been similarly associated with rapid infant growth.^48^ Although fungal abundance in the gut at one year of age has been inversely correlated with reduced BMI and increased height in Norwegian children,^52^ the role of fungi in early life on subsequent childhood growth and OW/OB outcomes remains poorly understood. To our knowledge, our study is the first to identify fungal biomarkers in early infancy, including depletions in *Malassezia* and an enrichment in *Rhodotorula*, which are correlated with increased childhood OW/OB risk and that are distinct from fungi associated with obesity in human adults.^53^ Further mechanistic studies will help elucidate whether such fungal differences in early life contribute to the altered growth trajectories.

Our findings indicate that accelerated gut microbiome functional and metabolic maturation evident at one month of age increases risk of OW/OB phenotypes at age two years, suggesting that interventions in very early-life may be necessary to alter disease course. Factors associated with higher-risk gut microbiomes at 1-month of age in our United States-based cohort included several correlates of lower socioeconomic status, including single parents with a shorter duration of formal education and reduced breastfeeding rates, which have previously been associated with childhood obesity^54,55^ and with structural racism.^56,57^ Thus, strategies including support for breastfeeding and addressing social inequities due to structural racism, a fundamental determinant of health,^58^ could decrease the prevalence of childhood obesity.

Pre- and post-natal exposures, including postnatal nutrition play a key role in shaping both host-mediated metabolism and infant intestinal microbiome functional capacity and metabolic productivity.^14,15,20,59^ Human milk is a complex, dynamic functional food that varies across individuals and with length of lactation to support nutritional requirements throughout infancy.^60^ Bioactives in breast milk, such as lactoferrin and immunoglobulin-A, regulate early-life microbial colonization in the gastrointestinal tract^61,62^ while nutritional substrates further select for co-evolved microbes capable of their metabolism.^63^ In contrast, infant formula primarily provides nutritional substrates, thus failing to exert the same chronologically programmed selective pressures as breast milk on the developing microbiome. Interestingly, the relationship between the infant fecal microbiota and childhood obesity and overweight phenotypes persisted despite adjustment for a number of biologically relevant factors, including infant age and breastfeeding status. This indicates that while formula feeding associates with increased risk of childhood obesity, accelerated microbiome development in the infant intestine appears to represent an independent early-life marker of childhood obesity risk. This may be due to additional intrinsic factors recently associated with childhood disease development such as heritable microbes^64^ and/or early life epigenetic training of immune function^65^ that promote accelerated microbial accumulation trajectories.

Broad metabolic differences between higher- and lower-risk for obesity infant microbiomes were evident and supported by parallel changes in gene content, indicating that multiple parallel microbial mechanisms may contribute to childhood obesity risk. Notable differences in microbial strategies for energy harvest were evident in lower- and higher-risk infants. Nutrient excess is known to promote mitochondrial dysfunction, leading to obesity-related pathologies.^66^ Numerous alternative microbial strategies for energy harvest were observed in lower-risk infants who were more likely to have been breastfed. This suggests that breastmilk-mediated enrichment of diverse microbial nutrient utilization pathways in the nascent gut microbiome may protect against childhood obesity by regulating host access to nutritional substrates. Failure to develop such microbial nutrient utilization strategies in infancy could conceivably lead to energy disequilibrium in the intestine and long-term host exposure to excess nutrient availability, thus increasing obesity risk. Importantly, given the transition from a milk-based to solid food diet during the first two years of life, it is plausible that unhealthy eating habits during the introduction of complementary foods conflate microbiome-mediated risks for childhood adiposity.

The feces of higher-risk infants were relatively depleted of GABA, which regulates neuro-endocrine mediated body weight control,^35,36^ intestinal barrier integrity,^38,39^ and is produced by lactic acid bacteria,^67^ implicating altered neuro-endocrine signaling in higher-risk infants. Though somewhat limited by sample size, our *in vitro* studies found that the cell-free fecal products of high-risk infants induced expression of genes and pathways associated with enteroendocrine progenitor cells, including *PCSK1N* which regulates proteolytic cleavage of neuroendocrine peptide precursors.^41^ Nutrient sensing enteroendocrine cells regulate macronutrient absorption and produce hormones that control energy homeostasis, appetite, satiety, post-prandial glucose levels, systemic metabolism and body weight.^68,69^ Thus, the cell-free products associated with higher-risk fecal microbiomes, which may include nutritional substrates in various stages of digestion, induce enteroendocrine cell progenitors that may contribute to childhood obesity by increasing host capacity for early-life macronutrient intake and harvest.

More broadly, enterocytes exposed to cell-free products of higher-risk fecal microbiomes, which can include variably digested nutritional substrates and microbiome-derived metabolites, were characterized by transcriptional reprogramming of genes involved in inflammation, lipid handling, fatty acid oxidation, and reduced barrier function – all previously described gastrointestinal features of patients with diagnosed obesity.^70–74^ Our data also found evidence that the cell-free products of the higher-risk infant fecal microbiome reduced intestinal epithelial barrier function. Intestinal hyperpermeability is central to the pathology of obesity and is associated with increased microbial product translocation, visceral adiposity, insulin resistance and chronic low-grade inflammation in adult disease.^75–77^ More recently, viable microbial translocation to mesenteric adipose via epithelial barrier breach was shown to promote expansion of mesenteric adipose tissue around inflamed intestines in humans.^78^ Our functional confirmation that cell-free products of the high-risk infant fecal microbiome promoted enterocyte hyperpermeability supports both a pathologic role and an additional mechanism by which the higher-risk infant fecal microbiomes may contribute to childhood obesity development.

Notably, key early-life gut microbial colonizers synonymous with breastfeeding i.e. *Lactobacillaceae*, *Bacteroidaceae* and *Bifidobacteriaceae*, and associated metabolic products such as agmatine^79^, correlated with improved intestinal barrier integrity *in vitro* and reduced risk of obesity development. Very recent studies have demonstrated that a multi-strain *Lactobacillus* cocktail improves intestinal barrier dysfunction and blood glucose levels in obese db/db mice^80^ and that the *Lactobacillus*-derived metabolite phenyllactic acid protects against diet-induced obesity in young mice fed a high fat diet.^81^ However, since nutrition, gut microbiome activity, and intestinal barrier integrity collectively contribute to childhood obesity development, further studies delineating their dynamic interactions along an early-life developmental gradient coupled with obesity outcomes measured at later stages of life are necessary to fully elucidate the generalizability of such observations to human populations.

Our study is not without limitations, which include insufficient granularity on factors such as complementary feeding practices and physical activity that contribute to OW/OB outcomes. Given the dynamics of early life gut microbiome development, future studies with more complete data on diet, feeding habits, and physical activity should further examine these relationships into later stages of infancy. The current study focused on the 1-month fecal microbiome and the functional implications of their associated metabolites in a subset of samples due to cost and sample availability, which reduced power in the multi-omic analyses and enterocyte assays and excluded the ability to detect key longitudinal shifts requiring longitudinal multi-omic datasets. Nonetheless, evidence from our current study supports the need for future studies with high resolution and quality stool sample collection over the course of infancy and early childhood.

Our findings implicate very early-life fecal microbiome dysfunction in the developmental origins of childhood obesity development and uncover key exposures associated with lower socioeconomic status and early life nutrition that co-associate with disease development. Functional infant microbiome analyses coupled with *in vitro* assays provide evidence that multiple microbial mechanisms involving nutrient harvest, altered enteroendocrine signaling and epithelial barrier impairment underlie increased risk of disease in childhood. Thus, early-life approaches to foster appropriately paced microbiome development and metabolism may help prevent obesity development in later childhood.

## Materials and Methods

### Study population, subsample criteria of subjects for stool microbiome analysis and OW/OB definition

The Wayne County Health, Environment, Allergy and Asthma Longitudinal Study (WHEALS) is a United States birth cohort that recruited pregnant individuals (*n* = 1258) aged 21–49 years between August 2003 and November 2007.^82^ Persons were considered to be eligible if they lived in a predefined cluster of contiguous zip codes in Wayne County, Michigan (including the city of Detroit), had no intention of moving out of the area in the subsequent two years and provided informed written consent. For this study, we selected WHEALS children who had a stool sample collected during a 1- and/or 6-month home visit and had completed their 24-month clinic visit with height and weight measurements (*n* = 543 subjects; *n* = 756 samples). Targeted ages at stool sample collection were 1 and 6 months of age, though actual ages ranged from 1 to 11 months. Infant stool was collected by field staff during the 1- and 6-month home visits and transported promptly back to the lab on ice to slow down metabolic processes before storage at − 80°C. Banked samples were shipped to the University of California, San Francisco (UCSF) on dry ice, where they were also stored at − 80°C until processed. At the study’s 2-year clinic visit, trained field staff measured child height and weight. Overweight or obesity (OW/OB) at age 2 years was defined using the 2000 age and sex adjusted CDC growth charts^83^ as BMI at or above the 85^th^ percentile and normal BMI as BMI between the 5^th^ to < 85^th^ percentile.

Since age strongly influences microbiome composition during early-life, DMM modeling was only applied to samples that were collected within a standard deviation of the mean collection age for each home visit to control for age-specific microbiome differences. Samples were stratified by time of sample collection (*n* = 403, 1-month; *n* = 353, 6-month). In the DMM analytical dataset, stool specimens from the 1-month visit were collected at a mean ±1 standard deviation (SD) of 39 ± 19 days (*n* = 349, median age 35 days; range 21–58 days) and stool specimens from the 6-month visit were collected at a mean ±1 SD of 205 ± 33 days (*n* = 287, median age 206 days; range 174–238 days).

### DNA extraction

Fecal DNA was extracted from stool samples using the modified cetyltrimethylammonium bromide (CTAB) method previously used for fungal and bacterial biomarker sequence-based profiling.^28,84^ Briefly, 500 µl modified CTAB extraction buffer was added to 25 mg of stool in a 2 ml Lysing Matrix E tube (MP Biomedicals) prior to incubation at 65°C for 15 min. Samples were bead-beaten (5.5 m/s, 30 sec) in a Fastprep-24 (MP Biomedicals) and then centrifuged (16000 × g, 5 min) before the top aqueous phase was transferred to a 2 ml polypropylene 96-well plate (USA Scientific). A further 500 µl modified CTAB extraction buffer was added to each LME tube, similarly bead-beaten and centrifuged to collect a total of 1 ml aqueous phase per sample. After adding 1 ml of phenol:chloroform:isoamyl alcohol (25:24:1) to the collected aqueous supernatant, samples were centrifuged (3200 × g, 20 min, 4°C) and the resulting top aqueous phase was transferred to a new 2 ml polypropylene 96-well plate (USA Scientific). Polyethylene glycol/NaCl (2 v/v) was added to the collected aqueous supernatant and incubated at room temperature for 2 h. Samples were then centrifuged (3200 × g, 60 min, 4°C), washed with ice cold 70% EtOH and resuspended in 30 μl of TE buffer (Invitrogen).

### PCR conditions and library preparation for bacterial and fungal biomarker sequencing

The V4 region of the 16S rRNA bacterial gene was amplified using primers designed by Caporaso *et al* ^85^ PCR was performed in 25 μl reactions using 0.025 U Takara Hot Start ExTaq (Takara Mirus Bio Inc.), 1× Takara buffer with MgCl_2_, 0.4 pmol/μl of F515 and R806 primers, 0.56 mg/ml of bovine serum albumin (BSA; Roche Applied Science), 200 μM of dNTPs and 10 ng of gDNA. Reactions were performed in triplicate with the following: initial denaturation (98°C, 2 min), 30 cycles of 98°C (20 s), annealing at 50°C (30 s), extension at 72°C (45 s) and final extension at 72°C (10 min). Amplicons from technical triplicates were pooled and verified using a 2% TBE agarose e-gel (Life Technologies), cleaned up and normalized using SequalPrep Normalization Plates (Applied Biosystems), and quantified using the Qubit dsDNA HS Assay Kit (Invitrogen). Samples were pooled in equal moles (5 ng), purified using AMPure SPRI beads (Beckman Coulter), quantified using KAPA SYBR (KAPA Biosystems), denatured, and diluted to 2 nM, and 5 pmol was loaded onto the Illumina Nextseq cartridge with 40% (v/v) of denatured 12.5 pM PhiX spike-in control.

The internal transcribed spacer region 2 (ITS2) of the fungal rRNA gene was amplified using the primer pair fITS7 (5’- GTGAATCATCGAATCTTTG-3’) and ITS4 (5’-TCCTCCGCTTATTGATATGC-3’).^85^ PCR was performed in triplicate in 25 µl reactions with 1× Takara buffer (Takara Mirus Bio), 200 nM of each primer, 200 µM dNTPs, 2.75 mM of MgCl_2_, 0.56 mg ml^−1^ of BSA (Roche Applied Science), 0.025 U Takara Hot Start ExTaq and 50 ng of gDNA. Reactions were conducted under the following conditions: initial denaturation (94°C for 5 min) followed by 30 cycles of 94°C (30 sec), annealing at 54°C (30 sec), extension at 72°C (30 sec) and a final extension at 72°C (7 min). PCR amplicons were verified, purified, quantified, and pooled as described above for bacterial library preparation. ITS2 PCR was performed on all stool samples, which produced ITS2 amplicons in *n* = 186 1-month and *n* = 180 6-month samples; samples without fungal data had no detectable ITS2 amplicons. The amplicon library was purified, quantified, denatured, and diluted similar to the 16S amplicon library described above. 10 pmol of the ITS2 amplicon library was loaded onto the Illumina MiSeq cartridge with 25% (v/v) of denatured 10 pM PhiX spike-in control.

### Biomarker sequence data processing

Paired-end sequences were assembled using FLASH v1.2.7^86^ requiring a minimum base pair overlap of 25 bp and demultiplexed by barcode using QIIME v1.9.1.^87^ Quality filtering was performed using USEARCH v8.0.1623^88^ to remove reads with > 2 expected errors. Quality reads were dereplicated at 100% sequence identity, clustered at 97% sequence identity into operational taxonomic units (OTUs), filtered of chimeric sequences by UCHIME,^89^ and mapped back to resulting OTUs using UPARSE;^90^ sequence reads that failed to cluster with a reference sequence were clustered *de novo*. Taxonomy was assigned to the OTUs using the Greengenes v13_5 database.^91^ Sequences were aligned using PyNAST,^92^ and FastTree 2.1.3^93^ was used to build a phylogenetic tree. Resulting sequence reads were normalized by multiply rarefying to 60,000 reads per sample as described previously^28^ to ensure reduced data were representative of the fuller data for each sample.

Fungal sequences were quality trimmed (Q score, <25) and removed of adaptor sequences using cutadapt.^94^ Paired-end sequences were assembled, demultiplexed by barcode, clustered into OTUs at 97% identity and filtered of chimeras using similar methods as described for 16S amplicons. Taxonomy was assigned using UNITE v7.0.^95^ Resulting sequence reads were normalized by multiply rarefying to 1,000 reads per sample to ensure reduced data were representative of the fuller data for each sample.

### Prediction of microbiota age using random forests

Random forest models were used to regress the relative abundances of all 16S rRNA-derived bacterial OTUs in infant stool samples against their chronological age using *randomForest* in R as previously described.^31^ Default parameters were used with the following exceptions: ntree = 10,000, importance = TRUE. Tenfold cross-validation was performed using the *rfcv* function over 100 iterations to estimate the minimum number of features needed to accurately predict microbiota age. The features most important for prediction were identified over 100 iterations of the *importance* function, and a sparse model consisting of the 50 most important features was constructed and trained on a set of *n* = 255 normal BMI infants (*n* = 356 fecal samples) randomly selected from the larger normal BMI infant set (including 50% of normal BMI GMC1 infants [*n* = 54]). This model was validated in the remaining *n* = 54 normal BMI GMC1 infants, and then applied to all remaining GMC1–3 infants to predict microbiota age. Microbiota-for-age z-scores (MAZ) was computed as previously described,^31^ enabling comparisons of microbiota maturity as the metric accounts for differing variance in predicted microbiota age throughout infant development.

### Metabolomic profiling

Stool samples selected had the highest posterior probability of GMC membership and sufficient remaining material for paired metabolomic and metagenomic analyses, reflecting representative feeding practices (more exclusively breastfed infants in GMC1; more exclusively formula fed infants in GMC3) and balanced BMI classifications across GMCs. Selected stool samples (200 mg; *n* = 28 GMC1 (*n* = 15 Normal, *n* = 13 OW/OB) and *n* = 32 GMC3 (*n* = 15 Normal, *n* = 17 OW/OB); Supplementary Table S9) were provided to Metabolon (Durham, NC) for Ultrahigh Performance Liquid Chromatography/Tandem Mass Spectrometry (UPLC-MS/MS) and Gas Chromatography-Mass Spectrometry (GC-MS) using their standard protocol (http://www.metabolon.com/). Identified compounds were compared to Metabolon’s in-house library of purified standards, which includes more than 3,300 commercially available compounds.

### Metagenomic processing and data analysis

DNA was extracted from stool samples that had been metabolically profiled (Supplementary Table S9) using the modified CTAB methods described above – Extracted DNA was provided to the Vincent J. Coates Genomic Sequencing Laboratory at the California Institute for Quantitative Biosciences for library preparation and 150-bp paired-end sequencing on an Illumina HiSeq 4000 (www.qb3.berkeley.edu/gsl). Only samples with > 50,000 total raw reads were included in the data analysis (*n* = 43, indicated within parentheses in Supplementary Table S9). The median number of raw reads per sample was 13,541,440 (IQR 6,300,000). The median number of reads following Q15 quality trimming and filtering human DNA using Bbduk v38.73 (https://sourceforge.net/projects/bbmap/) was 13,367,212 (IQR 2,073,330). All analyses were performed on trimmed and filtered reads. HUMAnN2 v2.8.1^96^ was used to identify genes, level4ECs and functional MetaCyc pathways from the short-reads, and to normalize outputs into copies per million (CPM). MetaCyc pathways were aggregated into functional families as previously described.^20^ Zero-inflated compound Poisson regression (*MaAsLin2*^97^ package) was used to determine pathways that differed in relative abundance between GMCs. Significantly different pathways (*P <* 0.05) with corroborating metabolomic data were visualized using BioCyc’s Pathway Collage and overlaid with log_2_ FC paired level4EC and metabolite (see *Metabolomic profiling*) values.

### Caco-2 enterocyte RNA sequencing and data analysis

Fecal samples from 4 GMC1 Normal BMI, 1 GMC1 OW/OB, 5 GMC3 Normal BMI and 7 GMC3 OW/OB infants (biological replicates) that had undergone metagenomic and metabolic profiling were used to prepare cell-free fecal products; excluded samples from these groups had insufficient material. Stool samples were homogenized 1 g/ml in pre-warmed phosphate-buffered saline (PBS) containing 20% fetal bovine serum (FBS). Samples were vortexed, incubated (37°C, 10 min) and centrifuged (14,000 rpm, 30 min). Supernatant was filtered through a 0.2 μm filter before being used to treat Caco-2 enterocytes, which have previously been used as a model to study infant enterocyte function.^98,99^

RNA was extracted from Caco-2 enterocytes treated with 5% v/v cell-free fecal products using the RNAqueous kit (ThermoFisher) and quantified using Qubit RNA HS assay (ThermoFisher). Extracted RNA was sent to the Vincent J. Coates Genomic Sequencing Laboratory at the California Institute for Quantitative Biosciences for library preparation and 150-bp paired-end sequencing on an Illumina NovaSeq 6000 (www.qb3.berkeley.edu/gsl). Demultiplexed paired-end reads were quality filtered and Q20 trimmed, removed of PCR duplicates and Illumina adapters using *HTStream* (https://github.com/s4hts/HTStream) and aligned to the human genome (Hg38 release) using *STAR* ^100^ with ENCODE recommended parameters.^101^ Features were assigned to transcripts using *STAR* and normalized using *DESeq2*.^102^ Differential expression was evaluated using *DESeq2* genes with at least 20 reads per gene in respective sample groups using a two-sided FDR. Log_2_-normalized read counts were obtained from *DESeq2* package, genes were filtered for presence in 75% of samples per comparison group, top variable genes were identified by the coefficient of variance and used to calculate principal components of Euclidean distances. Differential gene expression was mapped onto WikiPathways (WP3942 and WP111) using *RCy3*.^103^ Gene set enrichment analysis of transcripts associated with intestinal epithelial cell states^104^ was performed using default recommended parameters. Gene set enrichment comparisons were controlled for FDR with two sided *P_FDR_* ≤ 0.05 results displayed.^105,106^

### Transepithelial electrical resistance (TEER) assays

Caco-2 cells were seeded at 20,000 cells/cm^2^ on 6.5 mm diameter transwell inserts with 0.4um pores for 18 days. Complete Minimal Essential Media (MEM) was changed every other day to maintain and polarize enterocyte monolayers. At Day 18 post-seeding, baseline TEER measurements were taken prior to cells being treated with serum-free complete MEM containing 5% v/v cell-free fecal extracts or control exposures. TEER measurements were assessed by first removing media in the bottom chamber of the transwell, followed by removal of media in the top chamber. Media was replaced by room temperature PBS (-/- Ca^2+^, -/-Mg^+^), and TEER was measured with an EVOM2 epithelial voltohmmeter (World Precision Instruments). TEER measurements were assessed at baseline and following 24 or 48 hours of exposure to fecal extracts using the procedure outlined above.

### Statistical analysis

Except where indicated, all analyses were performed using the R statistical programming environment. Faith’s phylogenetic diversity was calculated in QIIME and Student’s or Welch’s t-tests or Wilcoxon tests were calculated in R, depending on the data distribution. Distance matrices based on unweighted and weighted UniFrac,^107^ Bray-Curtis and Canberra algorithms were calculated in QIIME to assess compositional dissimilarity between samples and were visualized using PCoA plots in R and Emperor,^108^ PERMANOVA was performed using *Vegan:Adonis*^109^ to determine factors that significantly (*p* < 0.05) explained variation in microbiota and metabolic β-diversity.

Dirichlet Multinomial Mixture (DMM) modeling,^26^ which uses an unsupervised Bayesian approach to cluster samples, was used to identify clusters of subjects based on bacterial taxon relative abundance. Samples were stratified by time of sample collection (1-month, 6-month; *n* = 349 or *n* = 287, respectively), with rarefied counts collapsed at the genus-level to avoid extreme sparsity. The best-fitting DMM model was determined using the Laplace approximation to the negative log model evidence, testing up to 10 underlying microbiota classes. Each sample was assigned to a particular Gut Microbiota Class (GMC) based upon the maximum posterior probability of membership; GMCs were examined for good separation and interpretability.

Hypothesized potential confounders were *a priori* specified as those potentially being associated with both GMC and childhood obesity, but not within the causal pathway. These variables included maternal marital status, maternal age at birth, maternal BMI first recorded in pregnancy, maternal and paternal education, location of residence, prenatal antibiotic and antifungal use, prenatal household smoke exposure, mode of delivery, gestational age at delivery, birthweight z-score, race-ethnicity of child, breastfeeding status at time of stool sample collection, and age at stool sample collection. Final multivariable models were built by weighing the importance of inclusion based on preconceived hypotheses, examining individually adjusted models to see which variables impacted effect sizes the greatest, and taking into consideration sample size concerns. Unadjusted and adjusted risk ratios (RRs) and corresponding 95% confidence intervals were calculated for OW/OB using log-binomial regression with maximum likelihood estimation, using PROC GENMOD in SAS version 9.4. In cases of model non-convergence, Poisson regression with robust error variance was alternatively used. Linear regression was used to test if BMI Z-scores were significantly different between GMCs; note that BMI Z-scores were normally distributed and model assumptions were evaluated. To determine which OTUs differed in relative abundance between GMCs, unnormalized read counts were transformed using *DESeq2*^110^ to identify log-Fold Change (FC) enrichment and corrected for multiple hypothesis testing using BH FDR (*P*_*FDR*_ < 0.05). Taxon fold change in relative abundance between GMCs was log_10_ transformed for illustration on a phylogenetic tree using iTOL v5.6.1. Metabolites exhibiting significantly different (*P*_*FDR*_ < 0.05) scaled intensities between GMCs were illustrated using Cytoscape v3.7.2.^111^

## Supplementary Material

Supplemental Material

Microbiome_Childhood_Obesity_Figures_20230523_EPS.rar

MicrobiomeObesity2YO_SupplementaryTables_20230523.xlsx

## Data Availability

All raw sequences are deposited in the European Nucleotide Archive (Study PRJEB52295 and PRJEB13896) and in the SRA Bioproject PRJNA648818. All additional datasets and materials are available from the corresponding author upon reasonable request.
