# Supplementary material for: Precocious infant fecal microbiome promotes enterocyte barrier dysfuction, altered neuroendocrine signaling and associates with increased childhood obesity risk
Source: Gut Microbes. 2023 Dec 20;16(1):2290661. doi: 10.1080/19490976.2023.2290661 (PMC10761186; doi:10.1080/19490976.2023.2290661)
Supplement: Supplemental Material [file KGMI_A_2290661_SM6877.docx]

**Supplemental Figure Legends**

**Supplemental Fig. 1. Compositionally distinct gut microbiota classes (GMCs) in feces exist during the first year of life.**

**(a)** Dirichlet multinomial mixture (DMM) model identifies three compositionally distinct bacterial GMCs as the best model fit in very early life (n=349, range 21 – 58 days; median = 35). Model fit was based on the Laplace approximation to the negative log model where a lower value indicates a better model fit. Univariate regression models identify variables related to microbiome composition and obesity that significantly alter (P < 0.05 marked by asterisk) **(b)** BMI Z-scores and **(c)** OW/OB Relative Risk (RR) at 2-years old. **(d)** DMM model identifies four compositionally distinct bacterial GMCs as the best model fit later in infancy (n=287, range 174 – 238 days; median = 206). Model was constructed as defined in **a**. **(e)** GMC designation significantly explains the observed variation in bacterial β-diversity in later life samples (n = 287; PERMANOVA of Unweighted UniFrac distances, R^2^ = 0.15; P = 0.001). Each dot represents an independent fecal sample. **(f)** Bacterial richness (n=349; Kruskal–Wallis; P < 2e-16) and **(g)** phylogenetic α–diversity are significantly greater in GMC3 (n=349; Kruskal–Wallis; P < 2e-16). Two-sided Wilcoxon rank sum test for significance of pairwise comparisons in **f** and **g**. Boxplots indicated within violin plots represent the median (center), the 25th and 75th percentiles, and the smallest and largest values within 1.5 × the interquartile range (whiskers).

**Supplemental Data Fig. 2. Microbial signatures of fecal microbiome development over the first year of life.**

**(a)** Age-stratified taxa summaries (presented at the family level) of bacterial relative abundance (n = 756; number of participants per age group is provided above bars). **(b)** Age-stratified taxa summaries (presented at the genus level) of fungal relative abundance (n = 366; number of participants per age group is provided above bars). **(c)** Tenfold cross-validation indicates that 50 bacterial OTUs (dashed line) are sufficient for random forest predictions of the chronological age of normal BMI infants on the basis of microbiota composition. Data show mean ± s.d. computed over 100 iterations. **(d)** The 50 most informative predictors to the random forest model, ranked in descending order of their importance to model accuracy. These bacterial OTUs were included in the sparse model used to calculate MAZ. Data show mean ± s.e.m. computed over 100 iterations.

**Supplemental Fig. 3. GMC3 and GMC1 are metabolically distinct.**

GMC1 contains greater concentrations of **(a)** glycerol 3-phosphate, methylglyoxal degradation products **(b)** lactate and **(c)** pyruvate, and **(d)** bilirubin. GMC3 is enriched in **(e)** l-urobilinogen, **(f)** the GABA precursor glutamine and **(g)** the weak GABA agonist 5-aminovalerate. Each dot represents an independent fecal sample in **a-g**. P values are two-tailed, from Welch’s two-sided t-test FDR BH-adjusted for multiple comparisons; P_FDR_ < 0.05 considered significant.

**Supplemental Fig. 4. GMC3 and GMC1 microbiomes exhibit distinct functional capacities.**

Comparative analysis of microbial metabolic pathways by GMC status indicated that GMC3 (n=23) microbiota are enriched for pathways that degrade **(a)** glycerol and **(c-d)** pyruvate, whereas GMC1 (n=20) is enriched in pathways that degrade **(b)** methylgloxal. GMC1 is also enriched in capacity for synthesis of **(e)** L-phenylalanine, **(f)** arginine and polyamines, **(g)** thiamine and **(h)** phylloquinol, and for **(i)** allantoin degradation. Each dot represents an independent fecal sample. P values are two-tailed, from zero-inflated compound Poisson (ZICP) models. Boxplots show normalized abundance (nCPM) and indicate the median (center), the 25th and 75th percentiles, and the smallest and largest values within 1.5 × the interquartile range (whiskers).

**Supplemental Fig. 5. Cell-free fecal products of 1-month-old GMC3 infants who become OW/OB at 2 years or GMC1 infants with normal BMIs at age 2 years induce divergent transcriptional responses in Caco-2 enterocytes.**

**(a-b)** Principal-component analysis of Euclidean distances of top 10,000 variably expressed genes (by coefficient of variation) and **(c)** heat map of most significantly differentially expressed (log_2_ FC > |1|, P_FDR_ < 0.05) genes in Caco-2 enterocytes treated with cell-free fecal products from GMC1 Normal BMI (n=4), GMC3 Normal BMI (n=5), GMC1 OW/OB (n=1) or GMC3 OW/OB (n=7) subjects, as determined by RNA sequencing, indicate a transcriptional shift in enterocyte expression based on GMC class and 2-year OW/OB phenotypes. Pathway analysis indicates that enterocytes treated with cell-free fecal products of GMC3 OW/OB (n=7) infants exhibit altered expression of **(d)** PPAR signaling and lipid metabolism and **(e)** reduced expression of mitochondrial oxidative phosphorylation genes compared with cells treated with GMC1 Normal BMI cell-free fecal extracts (n=4). Genes in **d** and **e** with log_2_ FC > |1| and P_FDR_ < 0.15 are indicated within bolded solid black boxes. Each dot represents an independent fecal sample in **a** and **b**. PERMANOVA test was used for significance for **a** and **b**. DESeq2 was used to calculate pairwise significance using a two-sided FDR and log_2_ FC.

**Supplemental Fig. 6. Cell-free fecal products of GMC3 infants decrease enterocyte epithelial barrier integrity *in vitro*.**

**(a)** Fold change of transepithelial electrical resistance (TEER) values of Caco-2 cells treated with cell-free fecal products from GMC1 Normal and GMC3 OW/OB subjects over 24 hours of treatment (n =15; One-way ANOVA). **(b)** Human IL1b, **(c)** human IL8, **(d)** human CCL20 concentrations in basolateral supernatants from cell-free fecal product treated Caco-2 cells after 48 hours, measured by ELISA (One-way ANOVA). **(e)** Enterocyte TEER 48-hour fold-change and explains 14% of observed variation in infant fecal bacterial β-diversity (n = 15; PERMANOVA of Bray-Curtis distances). **(f)** Relative abundance of bacterial taxa significantly (P_FDR_ < 0.05) associated with TEER values. **(g)** Bar plot of fecal metabolites correlated with TEER values (P_FDR_ <0.1). For **a-e**, each dot indicates a biologically independent infant sample. For **f**, each dot represents one bacterial taxa. Fixed effects models were used to calculate significant bacterial taxa in **f** and Spearman correlation was used to associate metabolites with TEER values in **g**.
